# Genes, Gender, Environment, and Novel Functions of Estrogen Receptor Beta in the Susceptibility to Neurodevelopmental Disorders

**DOI:** 10.3390/brainsci7030024

**Published:** 2017-02-23

**Authors:** Mukesh Varshney, Ivan Nalvarte

**Affiliations:** Department of Biosciences and Nutrition, Karolinska Institutet, Huddinge 14183, Sweden; mukesh.varshney@ki.se

**Keywords:** estrogen, testosterone, dyslexia, aromatase, brain, neurodevelopment, sex-difference, hormone, BPA, epigenetics

## Abstract

Many neurological disorders affect men and women differently regarding prevalence, progression, and severity. It is clear that many of these disorders may originate from defective signaling during fetal or perinatal brain development, which may affect males and females differently. Such sex-specific differences may originate from chromosomal or sex-hormone specific effects. This short review will focus on the estrogen receptor beta (ERβ) signaling during perinatal brain development and put it in the context of sex-specific differences in neurodevelopmental disorders. We will discuss ERβ’s recent discovery in directing DNA de-methylation to specific sites, of which one such site may bear consequences for the susceptibility to the neurological reading disorder dyslexia. We will also discuss how dysregulations in sex-hormone signaling, like those evoked by endocrine disruptive chemicals, may affect this and other neurodevelopmental disorders in a sex-specific manner through ERβ.

## 1. Introduction

The development of the human brain requires a very fine-tuned orchestration of diverse spatial and temporal cues modulating a regulatory interconnected network. Even a slight miss-regulation may have neurodevelopmental consequences resulting in different outcomes, such as varying degrees of cognitive or psychiatric disorders. Many of these outcomes affect males and females differently, suggesting that chromosomal and sex-hormone effects may play important roles during development [1]. Males are more likely to develop autism spectrum disorders (ASD), attention deficit hyperactivity disorder (ADHD), schizophrenia, and dyslexia, whereas females are more likely to be diagnosed with depression and anxiety [2]. In addition, neurodegenerative diseases such as Parkinson’s disease (PD) and Alzheimer’s disease (AD) affect males and females differently, with differences in susceptibility, progression, and severity [3,4,5,6,7]. Although hereditary components exist, recent studies have pointed out, for example, much lower heritability towards ASD (about 50%) than previously considered [8,9]. This suggests that environmental factors may play a larger role than initially thought [2,10,11]. Sex-specific events, including altered sex-hormone signaling, during early brain development are emerging as key processes that can influence the susceptibility for these disorders. The time window of sex hormone actions is vital in view of the long-term effects on neuronal development. Broadly, two types of hormonal effects, organizational and activational, are believed to contribute to the etiology of neuronal disorders, of which organizational effects presumably ensue during early development. This is at a time when many of the neural structures are orchestrated and establish permanent changes in the brain. Since neurodevelopmental disorders typically persist with an early onset, any hormonal influence on the occurrence of these disorders is expected to be organizational [12]. These effects are paramount during sensitive periods representing the windows of time when a tissue can be formed [13]. Outside this sensitive period, usually postnatally, the effects of hormones are restricted in protecting the animal from disruptive effects. It implicates that even a slight dysregulation of hormone signaling during early development of the brain could manifest long lasting behavioral deficits, whereas such hormonal perturbations in the adult brain is not as devastating.

This short review aims at summarizing and discussing the advances achieved in understanding the signaling of the female sex-hormone estrogen during early brain development. We will focus on the estrogen receptor beta (ERβ) where its recently discovered effects on the epigenome, and sensitivity towards endocrine disruptive chemicals, may bring new concepts behind sex-hormone signaling during neurodevelopment. In particular, such knowledge may be of specific relevance in understanding the etiology of the several neurological disorders. One such is the reading disorder dyslexia, which we will use to illustrate how the recent advances in ERβ research may give clues to how genes, gender, and the environment may affect its susceptibility.

## 2. Chromosomal Effects: *SRY*

It is clear that sex hormones such as estrogen and androgens are needed for proper development and maturation of the brain [14,15,16]. In addition, sex chromosomal genes are also of importance. Several studies have shown that both the over- and under-dosage of sex chromosomes (e.g., copy number effects or escape from X-chromosome inactivation) is associated with neurological deficits regardless of gonadal phenotype [2]. This may be linked to over- or under-activation of genes involved in neurodevelopment located either at the X chromosome (e.g., *NLGN4X*, *USP9X*, *Xlr3b*) [17] or Y chromosome (e.g., *Dby*, *Eif2s3y*) [18]. In recent years, the Y chromosome-coded *SRY* gene (sex-determining region on the Y chromosome) has gained attention as a major factor in sex-specific brain development, where its dysegulation has been associated with neurological deficits (see Kopsida E. et al. for review [19]). *SRY* itself is responsible for the activation of genes important for male gonadal development and therefore for the production of gonadal testosterone, consequently leading to the masculinization of the brain and development of secondary male sex-characteristics [20,21,22,23]. Interestingly, *SRY* is also found in neurons, where it can directly regulate the transcription of the dopamine biosynthesis machinery (including *TH*, *MAO-A*, *DBH*, *DDC*, and *D2R*) and catecholamine-dependent functions in adult male dopaminergic neurons [18,24,25,26]. This may provide a genetic basis for the male preference for “fight-or-flight” sympathetic reactivity over female “tend-or-befriend” in response to stress [27]. In addition, PD and ADHD are more common in males than in females. It is therefore tempting to speculate that the increased prevalence in males could, to some extent, be explained by abnormal regulation or function of *SRY* on dopaminergic neurons. Since females lack *SRY*, other factors compensate, and one such factor may be estrogen. Nevertheless, although estrogen has ascribed neuroprotective effects [28], there are conflicting results on the neuroprotective benefit of estrogen administration to postmenopausal women with PD [29,30,31]. In this respect, a different developmental “high-wiring” of the female brain compared to the male brain could account for the lower prevalence of PD in females.

## 3. Estrogen Signaling during Brain Development

The female sex-hormone 17β-estradiol (E2), commonly referred as estrogen, is primarily produced in the ovaries and is responsible for female sexual development and regulation of the menstrual cycle. The steroidogenic pathway of E2 synthesis involves numerous enzymes to convert cholesterol to testosterone followed by aromatization of testosterone to E2 by the enzyme aromatase (gene name *CYP19A1*). In rodents, the fetal and perinatal brain is very well protected from circulating E2 (e.g., E2 coming from the mother or the placenta). This protection is achieved by the abundant plasma protein α-fetoprotein (AFP, a fetal analog of serum albumin), which binds and reduces the bioavailability of E2 [32]. In humans, AFP has low affinity for E2 [33] and its developmental function is less clear. Instead, the high-affinity steroid binding protein sex hormone-binding globulin (SHBG) may have a similar function to rodent AFP in humans and primates with the exception that it has higher affinity for androgens than E2, and may thus protect the female brain from masculinization by androgens [34,35]. Interestingly, in females the fetal ovaries do not produce E2 until after birth (in mice at day P7) [36], but they do produce androgens such as dihydrotestosterone (DHT), which is rapidly metabolized to the estrogenic steroid 5α-androstane-3β, 17β-diol (3βAdiol) [37]. In males, 3βAdiol is produced from DHT similarly in the immature testes, but at a later postnatal period (after P20 in rats) [38]. Since SHBG may bind a large portion of circulating androgens, such as escaping DHT, it may be the estrogenic effect of 3βAdiol that is the more relevant in the fetal developing brain, and as mentioned above, 3βAdiol is more abundant in the female perinatal circulation, suggesting sex-dimorphic effects of 3βAdiol during the perinatal period. In addition, the male and female brain is a major site for E2 production [39], and aromatase, a key enzyme in the last step of E2 synthesis, is widely expressed in the neurons of fetal and adult brain [40,41,42,43]. Knockout of aromatase in rodents does not give major neuronal abnormalities; however, these mice show lower sexual motivation, which can be rescued with exogenous E2 administration [44]. In fact, testosterone, and its receptor the androgen receptor, does not appear to be directly responsible for the perinatal masculinization of the brain and for male typical behavior. Instead, it is the local aromatase-dependent conversion of testosterone to E2 in the perinatal brain that accounts for these effects [45]. Local E2 biosynthesis in the fetal brain occurs late in gestation upon expression of brain aromatase [46,47]. Prior to this, 3βAdiol produced by the immature ovaries is the main circulating estrogen. The immature testes also produce 3βAdiol, but only at postnatal time points [38,48]. Female fetuses are therefore exposed to the estrogenic effect of 3βAdiol earlier than male fetuses. 

The two main receptors for E2 are the nuclear receptors estrogen receptor alpha (ERα) and beta (ERβ). Both receptors appear to be highly expressed throughout the fetal mouse brain [36,49,50] until postnatal day P7, after which their levels sharply drop. This drop coincides with the initiation of E2 production by the ovaries [36,51]. ER localization in the mature brain is restricted to specific brain regions and cells: ERα predominantly localizes to the regions involved in arbitrating sexual behavior such as hypothalamus, whereas ERβ has a broader distribution in neurons of hippocampus, cerebral cortex, dorsal raphe, substantia nigra, and amygdala, as well as in microglia and oligodendrocytes [49,52]. ERα mRNA in the mouse brain is observed form day E16.5 [50] and ERβ mRNA from day E10.5, with a peak in protein levels at day E18.5 [53]. It is likely that varying levels of ERs may be expressed even earlier as embryonic stem cells, embryoid bodies, and neuronal progenitors have been reported to express both ER subtypes [54,55,56]. Knockout of ERα (ERα-/-) or ERβ (ERβ-/-) in mice show different behavioral and brain morphological effects. ERα-/- mice display decreased aggressiveness and aberrant sexual behavior linked to hypothalamic and pituitary defects [57,58], whereas ERβ-/- mice have increased aggressive behavior [59,60]. The ERβ-/- mice display a defective migration and layering of cortical neurons as well as impaired spatial learning [53,61,62] and increased anxiety behavior, which appears to be independent of E2 administration [62,63]. Such behaviors have not been shown for ERα-/- mice, suggesting that ERβ is the main ER isoform in regulating neuronal development associated with cognitive and affective behaviors [49]. In addition, fetal 3βAdiol preferentially binds ERβ over ERα [64], strengthening the importance of ERβ in the developing brain. However, its mechanistic understanding here is far from clear. In adult rodents, it has been shown that ERβ is expressed in serotonergic neurons of the dorsal raphe nucleus, where it mediates E2-dependent tryptophan hydroxylase (TPH) production [65,66], the rate-limiting enzyme for serotonin synthesis, and maintenance of serotonergic neurons [67]. ERβ is also the main ER isoform in dopaminergic (DA) neurons of the substantia nigra [68]. It is well established that E2 promotes neuroprotection of these DA neurons [69,70,71,72]. However, it is not clear if it is a direct effect of E2 on these neurons or if E2 exert its neuroprotective effect through oligodendrocytes, microglia, or astroglia. Interestingly, both oligodendrocytes and microglia express mainly ERβ, while astrocytes express ERα [73,74]. Future studies with cell-specific ER knockout models will bring knowledge to which cell type and which ER subtype is responsible for the neuroprotective effects of E2 in the adult brain, as well as the observed behavioral deficits linked to neurodevelopment. 

## 4. A New Role for ERβ in Epigenetic Remodeling

Epigenetic remodeling events such as DNA methylation and histone modifications are crucial for regulating gene expression during embryonic development [75,76]. It is known that nuclear receptors can attract chromatin-remodeling coactivators with histone acetyltransferase (HAT) activities, promoting gene transcription [77]. DNA de-methylation can be achieved passively by inhibition of the DNA methylation maintaining enzyme DNA methyltransferase (DNMT) during DNA replication [78]. However, it can also be achieved by active DNA de-methylation through oxidation of methylated CpG (cytosine-guanine) marks on the DNA by ten-eleven translocation (TET) proteins and DNA dioxygenases [79]. The oxidized methylated cytosine at the CpG is then recognized by thymine DNA glycosylase (TDG) of the base excision repair system and replaced by an unmethylated cytosine [80,81]. Increased levels of oxidized methylated CpGs (the prerequisite for active DNA de-methylation) have been found in gene regulatory regions of neurons and pluripotent cells [82], coinciding with bivalent histone marks (H3K4m2/3 and H3K27m3) [83]. Active DNA de-methylation pathways have consequently been found to be working during embryonic development and differentiation [84,85]. Studies have shown that transcription factors may direct de novo DNA methylation at proximal regions [86,87,88]. However, the mechanism behind how DNA de-methylation is directed has been elusive. Several studies have shown that nuclear receptors may be involved in regulating DNA methylation [55,89,90,91,92,93,94]. With regard to neurodevelopmental sex-hormone signaling, it is particularly interesting that ERβ may have an important function in DNA de-methylation [55,93]. Recent studies have shown that ERα and ERβ can bind and direct TDG to gene regulatory regions [55,95], where a direct effect on DNA methylation of genes involved in embryonic development was shown for ERβ (not ERα) in mouse embryonic fibroblasts irrespective of ligand treatment [55]. Thus ERβ may function at different levels during the neurodevelopment: by directly promoting transcription of target genes in a ligand-dependent or -independent manner, or by ligand-independently directing DNA de-methylation machineries to different gene regulatory regions during early development. It is not known yet if the latter also takes place during later developmental and mature stages. However, these data put forward ERβ as a very important factor regulating embryonic development. Surprisingly, ERβ-/- mice do survive with relatively mild morphological defects [53,96], suggesting that compensatory mechanisms exist (e.g., through ERα) or that ERβ may exert its effects during short developmental windows. Such windows could be very sensitive to disruptions in endocrine homeostasis.

## 5. Endocrine Disruptors and Neurodevelopment

Endocrine disruptive chemicals (EDCs) are environmental compounds that have endocrine active properties and can modulate the function of steroid hormone receptors. Such modulations may either be agonistic or antagonistic depending on receptor type and dose. Two types of EDCs are bisphenol A (BPA) and diesters of 1,2-benzenedicarboxylic-acid (phthalates), which have received attention due to their vast use as plasticizers in common plastic products [97,98]. As discussed above, sex-hormone signaling is very important for neuronal development. Therefore, *in utero* and perinatal exposure to EDCs such as phthalates and BPA may cause imbalanced sex-hormone signaling that may affect neuronal development. Recent biomonitoring data show that exposure to EDCs has increased during the past decades [99]. This coincides with a remarkable increase in children with neurodevelopmental disorders attending child and adolescent psychiatric clinics and increase in ASD prevalence worldwide, even when correcting for new diagnostic practices and increased attention to the hallmarks of ASD [8,100]. BPA interfere with the estrogen signaling [101,102] and phthalates with the androgen and estrogen signaling [103]. Their exposure during development has been associated with neuronal and behavior disorders, as well as affecting the reproductive system [104,105,106,107,108]. Like many other EDCs, BPA has a non-monotonic dose response curve, giving rise to debates regarding the safe dose and its agonistic or antagonistic properties on the estrogen receptors [109]. What is clear so far is that developmental exposure to BPA in various in vivo models results in anxiety, lasting cognitive deficits, and behavioral abnormalities, and that noted findings can be associated with behavioral outcomes in BPA-exposed children (for systematic review see Ejaredar M. et al. [110]). Interestingly, several in vivo models have shown that BPA also has sexually dimorphic effects on brain functions [111,112,113,114,115,116,117] and epidemiological studies have also shown differences between BPA exposed boys and girls regarding neuropsychiatric outcomes [118,119,120]. Recently it was demonstrated that perinatal exposure of male rats to low, physiological levels of BPA altered their stress response through dysregulation of ERβ [121], and that this effect could be linked to ERβ’s role in DNA de-methylation [55,121]. It was shown that BPA was equally potent as the anti-estrogen ICI 182,780 in inhibiting ERβ’s recruitment to the gene regulatory region of *Fkbp5*, a gene involved in the negative feedback of glucocorticoid signaling and stress response, and that this resulted in increased DNA methylation and decreased *Fkbp5* gene expression in an ERβ-dependent and sexually dimorphic manner [121]. In combination with the data discussed above, suggesting that ERβ may affect the epigenetic control of genes involved in embryonic development [55], dysregulation of ERβ by EDCs such as BPA may have neurodevelopmental consequences that deserve deeper investigation.

## 6. Sex Hormones and Dyslexia Susceptibility

Dyslexia is a neurodevelopmental disorder characterized by difficulties in reading and spelling, despite normal intelligence. Around 5% of children and adolescents suffer from dyslexia and it is more prevalent in boys than girls, especially when in combination with comorbidities such as ADHD [122,123]. This sex bias has been debated and some epidemiological studies have found that males are up to 4 times more likely to be diagnosed with dyslexia compared to females, whereas another study found no sex differences (for review see [124]). Clearly, more studies are warranted to accurately determine the sex difference, taking the environment and co-occurrence of neurodevelopmental and psychiatric disorders into account. Although there is no cure, defining the severity of dyslexia and treatment of possible concurrent mental disorders, and regular individual spelling and reading support in school, has been successful in minimizing the symptoms later in life [123]. Dyslexia has a heritable component associated with several candidate genes restricted to specific risk loci on different chromosomes [125,126]. However, an environmental component appears to exist, which remains virtually uncharacterized [122]. Two proposed candidate genes located on chromosome 15q21 that may bring clues to an environmental link to the susceptibility of dyslexia are *CYP19A1* and *DYX1C1* [127,128]. *CYP19A1*, encodes, as mentioned, the aromatase enzyme involved in conversion of testosterone to E2 and in sexual maturation of the brain [44,45]. A dysregulated aromatase gene could result in androgen production without conversion to E2 and thus the possibility for increased androgen exposure of the brain. Interestingly, high testosterone levels during the perinatal period has been proposed as a potential risk factor for learning disabilities and ADHD, and may impact on neuronal development [129,130]. This is consistent with observations that testosterone increases neural lateralization by promoting apoptosis in the right brain hemisphere while slowing development of the left hemisphere [129,131]. Although they are different neurological disorders, an altered hemispheric lateralization has been observed in both ADHD patients [132] and in dyslectic children [133]. It is tempting to speculate that an increased testosterone level in these individuals is a result of dysregulated local E2 production due to aberrant *CYP19A1* expression. In this respect, male fetuses would be more affected since females produce the estrogenic 3βAdiol by the fetal ovaries, and would thus be more protected from loss of local E2 production. 

**Figure 1:**
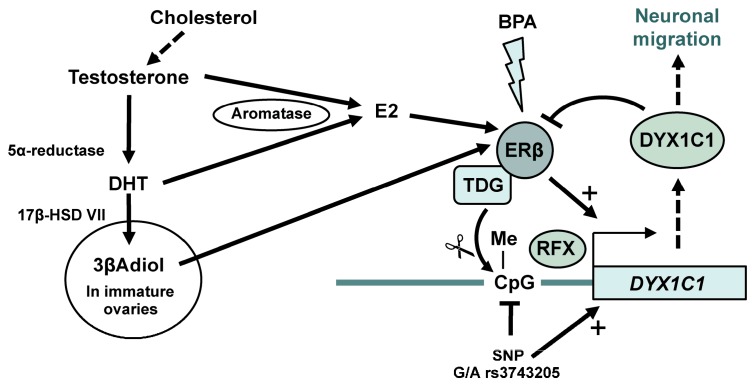
A model of how 17β-estradiol (E2) and 3βAdiol production may affect ERβ regulation of dyslexia susceptibility 1 candidate gene 1 (*DYX1C1*) gene expression during neuronal development. E2 is produced locally through the conversion of androgens to E2 by the enzyme aromatase (*CYP19A1* gene). In the normal situation, ERβ can promote DYX1C1 expression E2-dependetly, but probably also by recruiting Thymine-DNA Glycosylase (TDG) to the promoter to replace methylated cytosines by unmethylated ones, thereby promoting *DYX1C1* transcription (+). A specific single nucleotide polymorphism (SNP rs3743205) may abolish this DNA methylation, loosing one important level of regulation. Indeed, the different levels of regulation of *DYX1C1* (including feedback degradation of ERβ) suggest that this gene must be under a very tight regulatory control, which would be disrupted by the SNP. If the second dyslexia candidate gene, *CYP19A1*, encoding aromatase, is dysregulated, it should result in lower local production of E2, leading to androgen accumulation. The developing female brain may be less affected by the loss of aromatase since the immature ovaries produce the estrogenic 3βAdiol, which can preferentially bind and activate ERβ. Finally, the complex regulation of *DYX1C1* may be very sensitive to hormonal imbalances such as those evoked by endocrine disruptive chemicals (EDCs) (e.g., bisphenol A, BPA) that can dysregulate ERβ’s transcriptional activity as well as ERβ-mediated DNA de-methylation events.

Postmortem human dyslectic brains show morphological brain abnormalities, such as cortical ectopias and heterotopias and that such abnormalities are more common in male dyslexic brains compared to dyslexic female or control brains [124,134,135,136,137]. These abnormalities are very similar to those found in rats with impaired prenatal *DYX1C1* expression. However, sex differences were not analyzed in these studies [138,139,140]. Although not entirely characterized, the function of *DYX1C1* appears to involve migration of cortical neurons [138,140,141] through modulating the assembly of ciliary structures [142]. Interestingly, E2 may be involved in *DYX1C1* expression. As previously mentioned, E2 is produced through aromatization of testosterone by the proposed dyslexia candidate gene aromatase (*CYP1A1*) [127]. ERβ (not ERα) was found to associate with *DYX1C1 cis*-regulatory region to promote its transcription in the presence of E2 [143]. *DYX1C1* protein itself then associates with ERs to promote their proteasomal degradation [144], implying that *DYX1C1* is under a tight feedback regulation. The transcription of *DYX1C1* also appears to be under tight epigenetic control, as methylation of a single CpG in its *cis-*regulatory region drastically reduced the ERβ-mediated transcription [143]. Interestingly, a single nucleotide polymorphism (SNP rs3743205 [G/A]) that was previously shown associated with dyslexia [139] lies within this CpG. Hence, this SNP impacts on the regulatory control of *DYX1C1* [143]. As described above, ERβ can direct DNA de-methylation events to specific loci [55,121]. This could also be the case for ERβ’s association at the *DYX1C1* regulatory region. Recent data show that ERβ deficiency in mouse embryonic stem cells correlates with decreased *DYX1C1* expression as long as the cells remain in their pluripotent stage [55]. More differentiated cells had higher overall DNA methylation and either less *DYX1C1* expression irrespective of ERβ status, or increased *DYX1C1* expression in the absence of ERβ, depending on cell type. This implies that *DYX1C1* expression is under a strict developmental and cell-specific control and can under some developmental windows be regulated by E2 through ERβ, including possibly by ERβ-mediated DNA de-methylation (Figure 1). Interestingly, a functional X-box motif is located at the same regulatory region to which ERβ binds upstream of the *DYX1C1* transcription start site [145]. X-box motifs bind regulator function X transcription factors (RFX TFs) that are involved in regulation of, among other, ciliary genes [146] and reading of DNA methylation marks [147,148]. Although an interaction between ERβ and RFX TFs has not yet been shown, differential methylation of RFX1 and RFX2 binding sites was correlated to the absence or presence of ERβ [55], further strengthening the hypothesis that ERβ has an important regulatory role on *DYX1C1*. Combined, these data suggest that the tight temporal and spatial control of *DYX1C1* expression is very sensitive to endocrine imbalances, such as those evoked by aromatase dysregulation and EDCs. As mentioned above, ovarian 3βAdiol may compensate for loss of E2 upon aromatase dysfunction in females, but obviously not in males. However, endocrine imbalances evoked by EDCs such as BPA may disrupt ERβ-mediated DNA de-methylation events [121] in both sexes, and may impact on *DYX1C1* expression (Figure 1).

Of note, the genomic region 15q in which *DYX1C1* and *CYP19A1* are located also contains several susceptibility genes for ADHD, a comorbidity of dyslexia [149]. Although no clear support exists for *DYX1C1* and *CYP19A1* being risk genes for ADHD, abnormal perinatal testosterone levels has been suggested to be a risk factor of ADHD [129,130]. In fact, low *CYP19A1* expression and abnormal testosterone levels have been observed in an ADHD rat model [150]. With this in mind, it may be speculated that abnormal perinatal testosterone levels (or defective testosterone aromatization to E2) could dysregulate perinatal *DYX1C1* expression and be predisposal to ADHD in addition to dyslexia. Such effects may not necessarily be visible at genomic level. Future studies will give us clues to the regulations and functions of *DYX1C1* and *CYP19A1* in ADHD patients. Thus, neurodevelopmental effects of sex hormones, ERβ, and EDCs must be considered as important new factors that need to be taken into consideration when studying the etiology of, and sex differences in, dyslexia and its comorbidities.

## 7. Conclusions

Males and females are affected differently by different neurodevelopmental disorders such as dyslexia, ADHD, and ASD. Clues to understanding these disorders may lie within their observed sexually dimorphic effects. These dimorphic effects may be ascribed to chromosomal differences, such as defective *SRY* expression, or to differences in sex-hormone signaling. New studies have suggested that ERβ may play a more important role in mediating both E2- (or 3βAdiol) dependent and independent signaling during neuronal development, as well as having new functions in regulating temporal DNA methylation dynamics. Such effects of ERβ may be dysregulated by exposure to EDCs, which are increasingly more present in our environment and correlate with neurological deficits. With this in mind, the neurological reading disorder dyslexia may be a prime example of how genes, gender, and environment may contribute to its susceptibility. A genetic component is established, with candidate genes such as *CYP19A1* and *DYX1C1* among others; however, environmental and sex-specific factors are likely to be involved too. In particular, imbalanced sex-hormone signaling, for example through aberrant *CYP19A1* expression itself or from environmentally present EDCs, could affect ERβ function and its regulation of *DYX1C1* expression. The recent discovery that ERβ may modulate DNA de-methylation further strengthens the importance of ERβ and a balanced sex-hormone signaling during development. In addition, males may be more affected by sex-hormone imbalances than females since the circulating estrogenic compound 3βAdiol, produced by the immature ovaries, may likely compensate for perinatal E2 dysregulations and thereby protect the female brain. Thus, understanding the interplay between genes, gender and environment may hold great promises in understanding and developing sex specific treatments or prevention strategies for neurological disorders.
